# The miR-545/374a Cluster Encoded in the *Ftx* lncRNA is Overexpressed in HBV-Related Hepatocellular Carcinoma and Promotes Tumorigenesis and Tumor Progression

**DOI:** 10.1371/journal.pone.0109782

**Published:** 2014-10-09

**Authors:** Qi Zhao, Tao Li, Jianni Qi, Juan Liu, Chengyong Qin

**Affiliations:** 1 Department of Gastroenterology, Provincial Hospital Affiliated to Shandong University, Jinan, China; 2 Department of Infectious Diseases, Provincial Hospital Affiliated to Shandong University, Jinan, China; 3 Central Laboratory, Shandong Provincial Hospital affiliated to Shandong University, Jinan, China; University of Hong Kong, Hong Kong

## Abstract

Hepatitis B virus (HBV) infection is a major risk factor for hepatocellular carcinoma (HCC). Previous studies have shown several long noncoding RNAs (lncRNAs) play various roles in HCC progression, but no research has focused on the expression pattern of microRNA clusters encoded in lncRNAs. The Ftx gene encodes a lncRNA which harbors 2 clusters of microRNAs in its introns, the miR-374b/421 cluster and the miR-545/374a cluster. To date, no research has focused on the role of the miR-545/374a and miR-374b/421 clusters in HBV-related HCC. In this study, 66 pairs of HBV-related HCC tissue and matched non-cancerous liver tissue specimens were analyzed for the expression of the Ftx microRNA clusters. Our results showed that the miR-545/374a cluster was upregulated in HBV-HCC tissue and significantly correlated with prognosis-related clinical features, including histological grade, metastasis and tumor capsule. Transfection studies with microRNA mimics and inhibitors revealed that miR-545/374a expression promoted in vitro cell proliferation, cell migration and invasion. The wild-type HBV-genome-containing plasmid or full-length HBx protein encoding plasmid was transfected into the Bel-7402 cell line and observed for their influence on miR-545/374a expression. We found that transfection of the HBV genome or HBx alone resulted in an increase in miR-545/374a expression. Next, by monitoring the expression of sera miR-545/374a before and after surgical tumor excision, we found serum miR-545/374a was tumor-derived and exhibited a sharp decrease 25 days after tumor excision. We also examined the gender-based difference in miR-545/374a expression among HCC patients and utilized microRNA target prediction software to find the targets of miR-545/374a. One of these targets, namely estrogen-related receptor gamma (ESRRG) was inversely correlated with miR-545 expression. In conclusion, the overexpression of miR-545/374a cluster located in the Ftx lncRNA is partially responsible for a poor prognosis, and monitoring sera levels of miR-545/374a may be a useful diagnostic marker for HCC.

## Introduction

Hepatocellular carcinoma (HCC) is one of the most frequently occurring cancers worldwide. A variety of risk factors are associated with HCC development, including the following most common risk factors: hepatitis B virus (HBV) infection, hepatitis C virus (HCV) infection, consumption of food contaminated with aflatoxin B1, heavy alcohol intake, and nonalcoholic fatty liver disease [1]. Due to the growing incidence of HCC, intense research efforts are currently being undertaken to understand the cellular and molecular mechanisms of the disease so that novel diagnostic markers and therapeutic strategies can be developed.

The mechanism of HBV induced oncogenesis may involve integration of viral genomic fragments into the human genome. An alternative hypothesis is that HBV produces regulators which participate in cellular pathways that lead to carcinogenesis. However, the exact mechanism by which HBV infection leads to HCC still remains obscure [1]. In this study, we provide evidence that overexpression of a microRNA cluster promotes tumorigenesis and tumor progression in HBV-related HCC.

RNA has become widely suspected as the culprit behind almost every case of epigenetic regulation in a variety of diseases. Only 1% of the mammalian genome carries protein-coding potential, yet 70 to 90% is transcribed to produce a large transcriptome of long noncoding RNA (lncRNA, defined as RNA>100 nucleotides in length) [2]. Clear roles have emerged for several lncRNAs which participate in HCC disease progression [3], [4]. Mechanistically, lncRNAs target a site-specific pathway, and may directly interact with chromatin/mRNAs and recruit histone-modifying enzymes to induce epigenetic silencing of target genes [3], [5], [6]. Interestingly, few studies have reported that some of these self-functional lncRNAs are also acting as microRNA (miRNA) precursors. The lack of experimental data on these miRNAs leaves open questions about their possible relation to malignant tumors.

**Figure 1:**
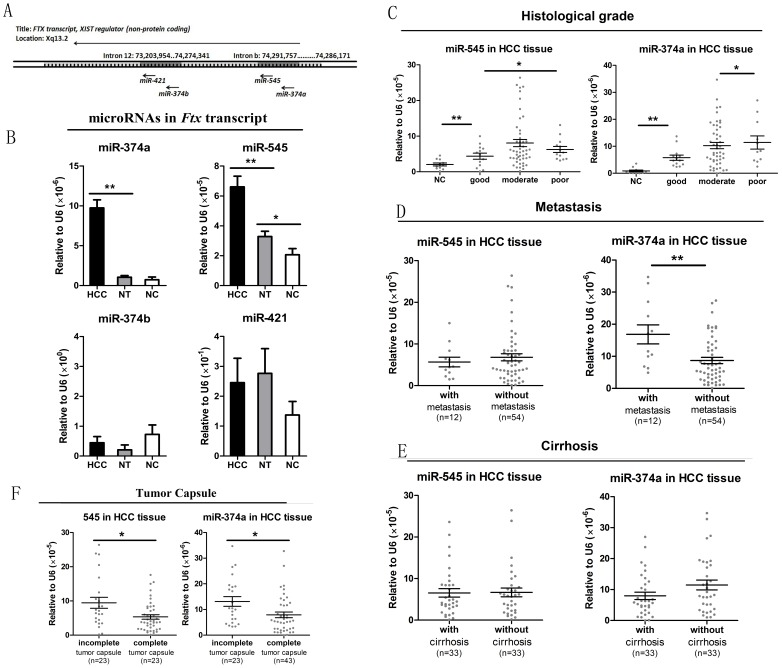
Expression pattern of microRNA clusters within the *Ftx* transcript and their correlation with key clinical features. (A) Genomic location of the Ftx microRNA clusters. Ftx contains a cluster of two microRNAs (miR-374b and miR-421) within intron 12 and a related cluster of two microRNAs (miR-374a and miR-545) within intron b. (B) Expression status of Ftx microRNA clusters (miR-374a, -545, -374b, -421) in human HBV-related hepatocellular carcinoma tissue (HCC; n = 66), matched distal non cancerous liver tissue (NT; n = 66) and negative control (NC; n = 11). The levels of each microRNA were determined by qRT PCR of tissue specimens. The error bars in B represent mean±Std. error. *P<0.05, **P<0.01, t test. (C-F) Analysis of individual microRNA expression patterns with well established prognosis-related clinical features. The levels of each microRNA was determined by qRT PCR of tissue specimens. Pathological examination included histological grade, metastasis, cirrhosis, and tumor capsule as described in the methods. The error bars in C-E represent mean ± Std. deviation. *P<0.05, **P<0.01, t test.

The *Ftx* transcript is a conserved functional lncRNA encoded within the X-inactivation center (*Xic*). *Ftx* encodes 4 microRNAs in its introns [7]. Intron 12 encodes 1 cluster of 2 microRNAs (miR-374b and miR-421), which is well conserved in different mammalian species. Intron b encodes 1 related cluster of 2 microRNAs (miR-374a and miR-545), which is absent in mouse and rat due to mutational changes (figure 1A). A recent study showed that miR-374a is upregulated in breast cancer tumors and promotes breast cancer metastasis by activating the Wnt/β-catenin signaling pathway [8]. It remains unknown whether the altered expression of miR-374a occurs independently from the other 3 microRNAs or whether the *Ftx* microRNA clusters are regulated as a group. No studies have explored the expression of the other 3 microRNAs and their potential relationship with malignant tumors. This raises a critical question that our research addresses: does *Ftx* microRNA clusters play a role in cancer progression?

Another example of altered microRNA expression occurs in hand-foot-and-mouth disease (HFMD) [9]. In this case miR-545 expression is upregulated, upon enterovirus 71 (EV71) or coxsackievirus 16 (CAV16) infection. Since viral infections can alter miR-545 expression, we explored the possible relationship between *Ftx* microRNA cluster expression and HBV infection.

In this study, the expression profiles of the miR-545/374a cluster and the miR-421/374b cluster derived from the *Ftx* transcript were examined in HBV-related HCC. Based upon correlation analysis with clinical features, the microRNAs were assayed for their ability to regulate tumor progression. *In vitro* cell culture assays were established to confirm the biological function of the microRNAs and potential regulating mechanism(s). Next, we followed a “tissue to serum, preoperative to postoperative” pattern to investigate the diagnostic value of corresponding serum microRNAs. Since *Ftx* plays a major role in mediating X chromosome inactivation, the influence of sex and the expression of sex related target genes were examined.

## Methods

### 1. Specimens

Sixty-six HBV-related HCC patients and 11 hepatic hemangioma patients (negative control) were recruited from February 2012 to January 2013 at the Provincial Hospital Affiliated to Shandong University. For each HBV-related HCC subject, 4 specimens were collected: (1) HCC tissue, (2) matched distal non-cancerous liver tissue, (3) 5 ml venous peripheral blood before surgery, and (4) 5 ml venous peripheral blood 20 days after surgery. Liver tissue and 5 ml venous peripheral blood were also collected from each negative control subject. For the use of materials for research purposes, written informed consent was obtained from each patient. The consent procedure and study protocol were approved by the Medical Institutional Ethical Committee of the Provincial Hospital Affiliated to Shandong University.

### 2. Clinical features

The samples obtained from HBV-related HCC subjects after surgery were analyzed by pathologists and features including histological grade, metastasis, tumor capsule and cirrhosis were reported. The levels of serum HBsAg and HBeAg were determined quantitatively by an electro-chemiluminescence immunoassay (ECLIA) on the Roche Elecsys 2010 immunoassay analyzer (Roche, Switzerland). The level of serum HBV-DNA was measured using the high-sensitivity fluorescent quantitative PCR Kit (DaAn Gene Co., China) and amplified in a PE 5700 fluorescence PCR apparatus (Perkin-Elmer, Norwalk, USA). α-fetoprotein (AFP), carcino-embryonic (CEA) and γ-glutamyltransferase γ(-GT) were measured by routine laboratory methods. Concrete information is shown in Table 1. No significant differences between the HBV-related HCC group and the negative control group with respect to age were found. However, there were significant differences concerning the gender of the patient and the levels of HBV-DNA, AFP, CEA and γ-GT (data not shown).

**Table 1:** Clinicopathological characteristics of patients with HBV-related hepatocellular carcinoma.

Characteristic	No. of patients (N = 66)
Age	
<50	25
≥50	41
Sex	
Male	45
Female	21
HBsAg	
Positive	55
Negative	11
HBeAg	
Positive	11
Negative	55
HBV-DNA	
≤1000	17
>1000	49
Cirrhosis	
Positive	33
Negative	33
Histological grade	
Good	13
Moderate	42
Poor	11
Metastasis	
with	12
without	54
Tumor capsule	
Complete	43
Incomplete	23
AFP(ng/ml)	
≤20	36
>20	30
CEA(ng/ml)	
≤10	53
>10	13
γ-GT(U/L)	
≤40	16
>40	50

HBsAg, hepatitis B surface antigen, HBeAg, hepatitis B e antigen, AFP, α-fetoprotein; CEA, carcino-embryonic antigen; γ-GT, γ-glutamyl transpeptidase, NA.

### 3. Cell culture

Human hepatocellular carcinoma cell lines Bel-7402, HepG2 (Keygen Biotech, Nanjing, China), and previously constructed stable HBV positive HepG2.2.15 cells were maintained in HyClone's media containing 10% fetal calf serum, 100 U/ml penicillin, and 100 mg/ml streptomycin at 37°C in a humidified atmosphere containing 5% CO_2_. Bel-7402 cells were seeded at a density of 2.0×10^5^ cells/mL and starved for 24 hours before transfection.

### 4. Plasmid transfection

The wild-type HBV genome-containing plasmid (pCDNA3.1-HBV), and the plasmid encoding full-length HBx protein (pIRSE-HBx) were transfected using Lipofectamine 2000 (Invitrogen, CA, USA) according to the manufacturer's protocol. Empty expression vectors were transfected as a negative control. Expression of plasmids in Bel-7402 cell lines was confirmed by high-sensitivity fluorescent quantitative PCR and western blotting.

### 5. Oligonucleotide transfection

MicroRNA 545 mimics, miR-545 inhibitor, miR-374a mimics, miR-374a inhibitor, and negative control duplex (designated as NC), were all synthesized by GenePharma (Shanghai, China). The sequences are listed in the **Table S1**. Oligonucleotide transfection was performed using Lipofectamine 2000 according to the manufacturer's protocol.

### 6. MicroRNA/mRNA extraction, reverse transcription, and real-time PCR analysis

The manufacturer's instructions were followed for each kit utilized. The mRNAiso Plus and microRNAiso Plus kits (Takara Bio, China) were separately used for mRNA and miRNA extraction from liver tissue. The MiRCURY RNA Kit (Exiqon, Denmark) was used for microRNA extraction from serum specimens. The PrimeScript RT Reagent Kit and the SYBR Premix Ex Taq (Takara Bio, China) were used separately for first strand cDNA synthesis and mRNA real time PCR [10]. The SYBR II PrimeScript miRNA RT-PCR Kit (Takara Bio, China) was used for microRNA reverse transcription and real time PCR. Beta-actin was used for normalization of mRNA, and U6 was utilized for normalization of microRNA. Specific and universal primers (Takara Bio, China) were used in pairs when amplifying microRNA. The primers are listed in the **Table S1**.

### 7. Western blotting

Bel-7402 cells were harvested after transfection with pCDNA3.1-HBx for 48 hours. Whole-cell extracts were prepared in lysis buffer as described previously [11]. Equal amounts of protein were subjected to electrophoresis on 10% SDS-polyacrylamide gels and transferred onto nitrocellulose membranes (Schleicher & Schuell BioScience GmbH, Dassel, Germany) by electroblotting. The membranes were incubated with rabbit anti-human HBx antibody (1∶1000 dilution; Abcam, Cambridge, Massachusetts, USA), followed by incubation with mouse anti-rabbit secondary antibodies (1∶5000 dilution, Abcam). Protein bands were detected using an enhanced chemiluminescence reagent (Sigma, USA).

### 8. MTT assay

Approximately 3×10^3^ Bel-7402 cells were plated in each well of a 96-well plate. After an overnight incubation, the cells were transfected separately with the NC, miR-545/374a mimics or inhibitor for 6 hours. Twelve hours after the medium was removed, colorimetric 3-(4,5-dimethylthiazol-2-yl)-2,5-diphenyl-tetrazolium (MTT; Sigma-Aldrich) was added to each well at various times following treatment. The 96-well plates were incubated at 37°C for 3 h. The plates were then centrifuged, and the formazan precipitates were dissolved in 100 ul of dimethyl sulfoxide [12]. The absorbance was measured at 490 nm using a Spectra Max M_2_ spectrophotometer (Molecular Devices, Sunnyvale, California, USA).

### 9. Cell invasion and migration assays

Cell migration and invasion were assayed using a transwell chamber (Millipore, USA) with and without Matrigel (BD, Franklin Lakes, USA), respectively. For the invasion assay, a transwell chamber was placed into a 24-well plate and was coated with 30 ul of Matrigel. In both transwell assays, Bel-7402 cells, 48 hours after transfection, were trypsinized and seeded in chambers at a density of 8×10^4^ cells per well. The cells were cultured in 200 ul of RPMI 1640 medium without serum, and 600 µl of RPMI 1640 medium with 10% FBS was added to the lower chamber. Sixty hours later, migrated cells were fixed with 100% methanol for 30 min. Non-migrated cells were removed by cotton swabs. Next, the cells on the bottom surface of the membrane were stained with crystal violet for 20 min. Cell images were obtained utilizing a phase-contrast microscope (Leica DM4000 B, Germany) [12].

### 10. Statistics

Data were analyzed with SPSS 16.0 statistical software (SPSS Inc., Chicago, Illinois, USA). The one-Sample Kolmogorov-Smirnov Test was used to confirm the normal distribution of experimental data. To evaluate significant differences between matched HBV-HCC tissue and nontumor tissue, matched serum specimens before and after surgery, paired t tests were performed. Unmatched continuous data were compared using an independent 2-tailed t test. Pearson's correlation (r) was used to measure correlation and logarithmic regression was utilized to derive the equation of the slope. In all cases, P<0.05 was considered statistically significant.

## Results

### The miR-545/374a cluster in the *Ftx* transcript is upregulated in HCC tissue and is significantly correlated with prognosis-related clinical features

To determine the status of the *Ftx* microRNA clusters, we measured the expression of individual microRNAs by quantitative RT-PCR in tissue specimens. As shown in figure 1B, the expression of the miR-545/374a cluster was lower than the miR-421/374b cluster, but showed clear variation in different groups. MicroRNA 545 was significantly upregulated in HCC tissue compared with non cancerous liver tissue (NT) (HCC = 6.60×10^−5^ vs. NT = 3.27×10^−5^; p = 0.003, paired t test), as well as in NT compared with negative control (NC) (NC = 2.06×10^−5^; p = 0.034, independent t test). Likewise, miR-374a expression was significantly higher in HCC tissue compared with NT (HCC = 9.73×10^−6^ vs. NT = 1.05×10^−6^; p = 0.000, paired t test), while no significant difference was found between NT and NC regarding miR-374a expression (NC = 0.73×10^−6^; p = 0.584, independent t test). On the other hand, the comparatively conserved cluster of miR-374b and miR-421 were highly expressed with large individual difference, but surprisingly showed no significant difference between 66 pairs of matched HCC tissue and corresponding NT. Furthermore, the expression levels of miR-374b and miR-421 did not show any differences between 66 HCC tissues and 11 NC. The result suggests that increased miR-545/374a cluster expression in the *Ftx* transcript may be an estimable event in human HBV-related HCC tumorigenesis.

To investigate the potential role of miR-545/374a in HBV-related HCC, the expression levels of these microRNAs were analyzed with key clinical features associated with tumor progression and disease prognosis. The histological differentiation analysis showed no restrict grade-by-grade variation. However, figure 1C shows that the expression of miR-545 is significantly higher in the poor differentiated group compared to the good differentiated group (p = 0.019), while the expression of miR-374a is significantly higher in the moderate differentiated group compared to the good differentiated group (p = 0.005). In addition figure 1F shows that, patients with an incomplete tumor capsule had higher levels of both miR-545 and miR-374a than patients with a complete tumor capsule (p = 0.04, p = 0.013). Furthermore, figure 1D shows that patients with distal metastasis had significantly higher miR-374a expression than patients without distal metastasis (p = 0.002). No significant differences were observed in the miR-545 and miR-374a expression levels with respect to cirrhosis (figure 1E). Likewise, no significant differences were observed for the levels of miR-545 and miR-374a with respect to age (figure S1A). The overall results indicate that high miR-545/374a expression may facilitate tumor invasion and distal metastasis, which may be responsible for a bad prognosis of HBV-HCC patients.

### The miR-545/374a cluster promotes HCC cell proliferation, cell migration and invasion

**Figure 2:**
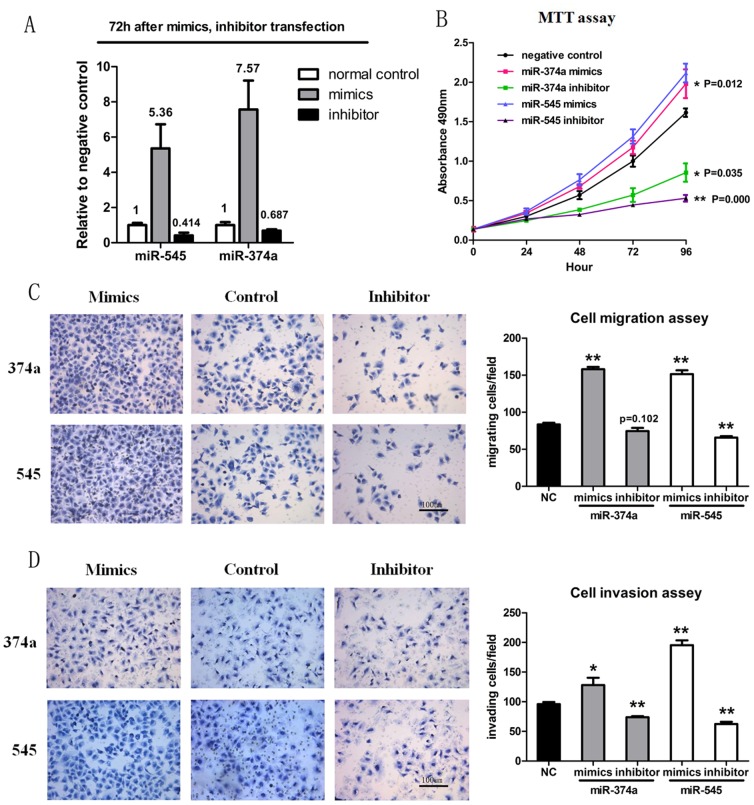
Functional analysis of miR-545 and miR-374a. (A) Expression status of miR-545 and miR-374a following transfection with microRNA mimics and inhibitors. The Bel-7402 cell line was transfected with individual microRNA mimics and inhibitors. Nonsense oligonucleotide was transfected as a negative control. The microRNA expression status at 72 h after transfection was monitored by quantitative RT-PCR. Experiments in A were repeated at least 3 times with similar results, and error bars represent mean ± Std. deviation. (B) Bel-7402 cells were transfected as described in figure 2A and a MTT cell proliferation assay was performed. Experiments in B were repeated at least 3 times with similar results, and error bars represent mean ± Std. deviation. (C) Bel-7402 cells were transfected as described in figure 2A and cell migration assays using transwell membranes were performed. Representative pictures of migration chambers, and average counts from five random microscopic fields are shown. Experiments in C were repeated at least 3 times with similar results, and error bars represent mean ± Std. deviation. (D) Bel-7402 cells were transfected as described in figure 2A and cell invasion assays using Matrigel-coated transwell membranes are shown. Representative pictures of invasion chambers, and average counts from five random microscopic fields are shown. Experiments in D were repeated at least 3 times with similar results, and error bars represent mean ± Std. deviation. *P<0.05, **P<0.01, t test.

To gain a mechanistic understanding of the biological significance of the upregulated miR-545/374a cluster, the Bel-7402 cell line was transfected with individual microRNA mimics and inhibitors. Nonsense oligonucleotide was transfected as a negative control. The microRNA expression status at 72 h after transfection was monitored by quantitative RT-PCR (Figure 2A). The inhibition rates for miR-545 and miR-374a were 58.6% and 31.3%, respectively.

To determine whether miR-545 and miR-374a have an impact on tumor cell proliferation, we transfected cells with various microRNA mimics and inhibitors (see above) and performed a MTT assay to monitor cell viability. As shown in figure 2B, the proliferation of HCC cells was significantly decreased after transfection with miR-545/374a inhibitors compared to negative control. Overexpression of miR-545/374a (via transfection of the mimics) in the HCC cell line promoted cell proliferation to a small degree. Next, transwell assays were performed using transfected cells (see above) to investigate the effect of miR-545/374a expression on cell migration (figure 2C) and invasion (figure 2D). Both microRNAs enhanced the migration and invasion ability of HCC cell lines, except that inhibition of miR-374a had no statistically significant influence in cell migration assays (P = 0.102). We also used wound healing assay to confirm the influence of miR-374a/545 expression on cell migration (Figure S2). Taken together, these results demonstrated that overexpression of the miR-545/374a cluster permits cell proliferation in the HCC cell line, and may exert a promoting effect on cell migration and invasion.

### Expression of miR-545/374a is positively regulated by HBV infection and may be induced by HBx expression

**Figure 3:**
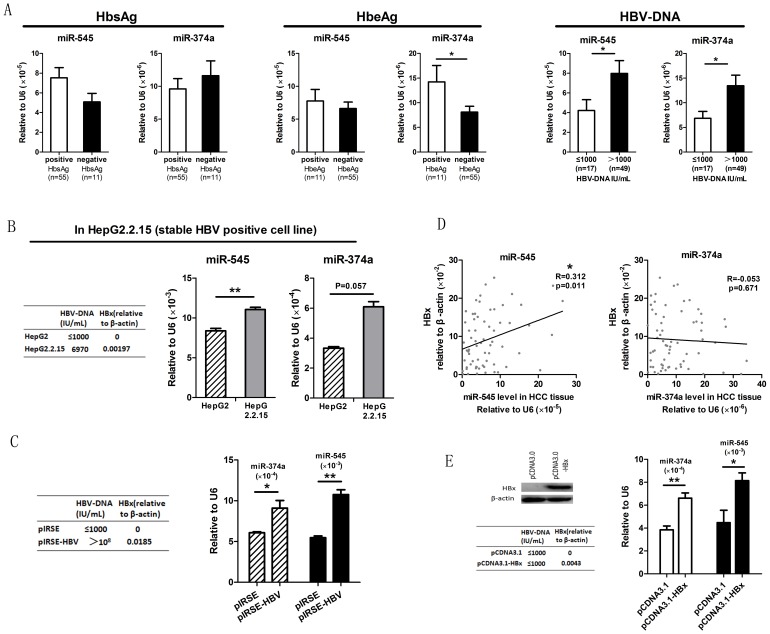
The influence of HBV infection on miR-545/374a cluster expression. (A) HCC subjects were grouped based on clinical HBV indices including HbsAg, HbeAg and HBV-DNA (Mean±Std.deviation, 10.64±9.158; Median, 7.035). The levels of miR-545 and miR-374a were determined by quantitative RT-PCR. Individual microRNA differences were measured by a student-t test between positive and negative groups. The error bars in A represent mean ± Std. deviation. (B) Comparison of microRNA expression levels between the stable HBV positive cell line HepG2.2.15 and its parental HCC cell line HepG2. The levels of miR-545/374a were monitored by quantitative RT-PCR in both cell lines. The error bars in B represent mean ± Std. deviation. (C) Analysis of microRNA expression levels in cells transfected with a HBV encoding plasmid. Cells were transfected with the HBV genome encoding plasmid (pIRSE-HBV) or the empty plasmid (pIRSE). To confirm the effective transfection of the HBV-genome containing plasmid we performed high-sensitive fluorescent quantitative PCR to detect HBV-DNA and quantitative real time PCR to detect HBx protein expression. The expression of miR-374a and miR-545 was measured by quantitative RT-PCR. *P<0.05, **P<0.01, independent t test. The error bars in C represent mean ± Std. deviation. (D) Analysis of miR-545/374a and HBx expression levels in HBV HCC subjects. The levels of miR-545/374a and HBx mRNA were assayed by quantitative RT-PCR. *P<0.05, Pearson's correlation. (E) Analysis of miR-545/374a expression in HBx overexpressing cells. Bel-7402 cells were transfected with a plasmid expressing HBx (pCDNA3.1HBx) or with an empty plasmid (pCDNA3.1). Western blotting and quantitative RT-PCR were used to confirm HBx protein expression after transfection. The levels of miR-545/374a were assayed by quantitative RT-PCR. The error bars in E represent mean ± Std. deviation. *P<0.05, **P<0.01, independent t test.

In light of previous evidence that the miR-545 expression profile can be altered by viral infection [9], we explored the possibility that overexpression of miR-545/374a might be related to HBV infection. Figure 3A shows that HBeAg positive patients showed a significantly higher level of miR-374a than HbeAg negative patients, and patients with a positive HBV-DNA level had higher expression of both miR-545 and miR-374a compared to patients with a negative HBV-DNA level.

To confirm our hypothesis that miR-545/374a expression might be related to HBV infection, we monitored the expression of miR-545/374a in HBV positive and negative cell lines. Quantitative RT-PCR was used to measure the miR-545/374a expression in the stable HBV positive cell line HepG2.2.15 and its parental HCC cell line HepG2 (figure 3B). A statistically significant increase in miR-545 expression was observed in the HepG2.2.15 cell line compared to the HepG2 cell line (p = 0.003), but the data obtained for miR-374a was not statistically significant (p = 0.057). Next, the HCC cell line Bel-7402 was transfected with the HBV-genome-containing plasmid (pIRSE-HBV) and empty pIRSE plasmid was utilized as a negative control. The levels of miR-545/374a were then monitored by quantitative RT-PCR. As hypothesized, figure 3C shows that both miR-545 and miR-374a were significantly increased after transfection of the HBV genome encoding plasmid (p = 0.001, p = 0.032 respectively).

HBx protein is a key regulator of HBV infection. Therefore, we monitored the expression of this protein in 66 HBV-HCC subjects and compared it to the levels of miR-545/374a. The levels of miR-545/374a and HBx mRNA were measured by quantitative RT-PCR. As shown in figure 3D, HBx expression is significantly correlated with miR-545 expression (r = 0.312, p = 0.011, Pearson's correlation). To investigate whether HBx acts as a potential regulator of the *Ftx* miR-545/374a cluster, an overexpression strategy was conducted via transfection of a HBx expressing plasmid (pCDNA3.1-HBx) into Bel-7402 cells. Figure 3E shows that the HBx protein was overexpressed in pCDNA3.1-HBx transfected cells. Furthermore, the expression level of miR-545/374a was increased in HBx expressing cells compared to negative control cells transfected with empty plasmid (p = 0.046, p = 0.008). Taken together, these data indicate that the expression of the *Ftx* miR-545/374a cluster is positively regulated by HBV infection, and HBx may be a key regulator in this process.

### Serum miR-545/374a are tumor-derived, and their expression are significantly decreased after surgical excision of tumor tissue

**Figure 4:**
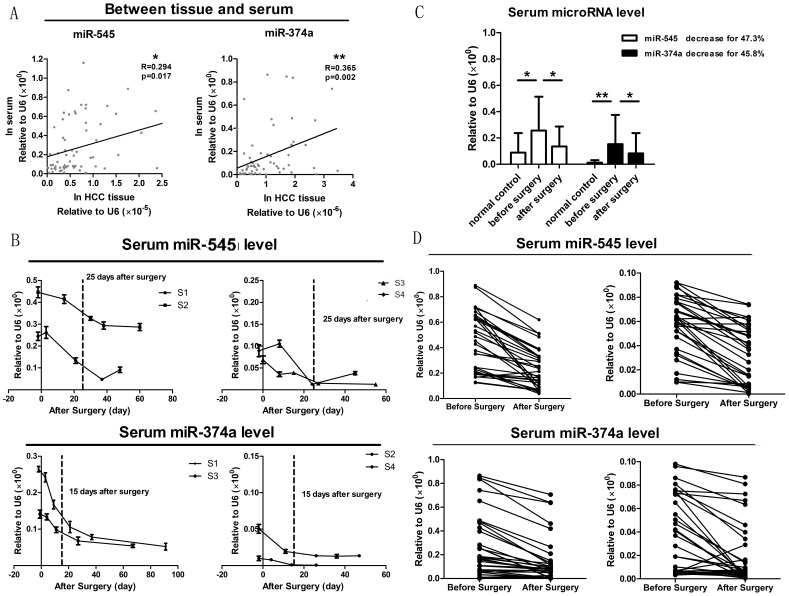
Tissue to serum analysis of miR-545/374a from the preoperative period to the postoperative period. (A) Tissue to serum analysis of miR-545/374a in HBV-HCC subjects. The levels of miR-545/374a in sera and tissue were assayed via quantitative RT-PCR. Pearson's correlations were calculated to measure microRNA expression between HCC tissue and serum. *P<0.05, **P<0.01. (B) Sera levels of miR-545/374a after surgical excision of tumor tissue. The sera from 4 representative HBV-related HCC subjects (S1, S2, S3 and S4) were collected at different time points after surgery. Quantitative RT-PCR was utilized to determine the levels of miR-545/374a in the sera samples. The dynamic levels of individual microRNAs in the sera were recorded as line charts. The error bars in B represent mean ± Std. deviation. (C) Pairwise comparison of microRNA levels between 66 pairs of preoperative sera and postoperative sera, and 23 negative control. The levels of miR-545/374a were measured by quantitative RT-PCR. The error bars in C represent Std. error. *P<0.05, **P<0.01, t test. (D) The microRNA expression variation from the preoperative period to the postoperative period is shown for each patient.

Tissue to serum analysis represents the process from mechanism to application. As a first step, the changing tendency of serum microRNAs should be tested to determine if their presence is consistent with their presence in tissue. Figure 4A shows the tissue to serum analysis of miR-545/374a in HBV-HCC subjects. Our study showed that, in 66 HBV-HCC subjects, the expression of miR-545/374a in preoperative period serum is significantly correlated with miR-545/374a expression in HCC tissue (r = 0.294, p = 0.017; r = 0.365, p = 0.002; Pearson's correlation). In addition, the expression of miR-545 and miR-374a both showed a statistically significant distinction between sera of HCC patients and sera of negative control patients (p = 0.030, p = 0.000, independent t test) (figure 4C). These data demonstrated that detection of miR-545/374a in serum may indicate HBV-related HCC occurrence.

To determine the levels of miR-545/374a in sera following surgical excision of tumor tissue, we collected 4 patients' sera repeatedly at different time points after surgery. The miR-545/374a levels were determined by quantitative RT-PCR. The dynamic tendency of individual microRNA levels in the sera samples were recorded as line charts (figure 4B). As the charts show, the sera miR-545 levels show a significant decrease at about 25 days after surgery, while the sera miR-374a levels decrease at approximately 15 days after surgery. Based on these data, we collected the postoperative sera more than 25 days after surgery. The miR-545/374a levels in the sera samples were determined by quantitative RT-PCR. Next, a paired t test was used to compare the microRNA levels between preoperative sera and postoperative sera (figure 4C). The results showed that the miR-545 concentration decreased by 47.3% (p = 0.001), while miR-374a similarly decreased by 45.8% (p = 0.042), which further confirmed that serum miR-545/374a is tumor-derived. Figure 4D shows the levels of miR-545/374a from the preoperative period to the postoperative period for each patient. As expected, nearly all patients show a reduction in miR-545/374a expression following surgery.

### Correlation analysis of preoperative serum miR-545 and miR-374a expression with prognosis/diagnosis related clinical features

**Figure 5:**
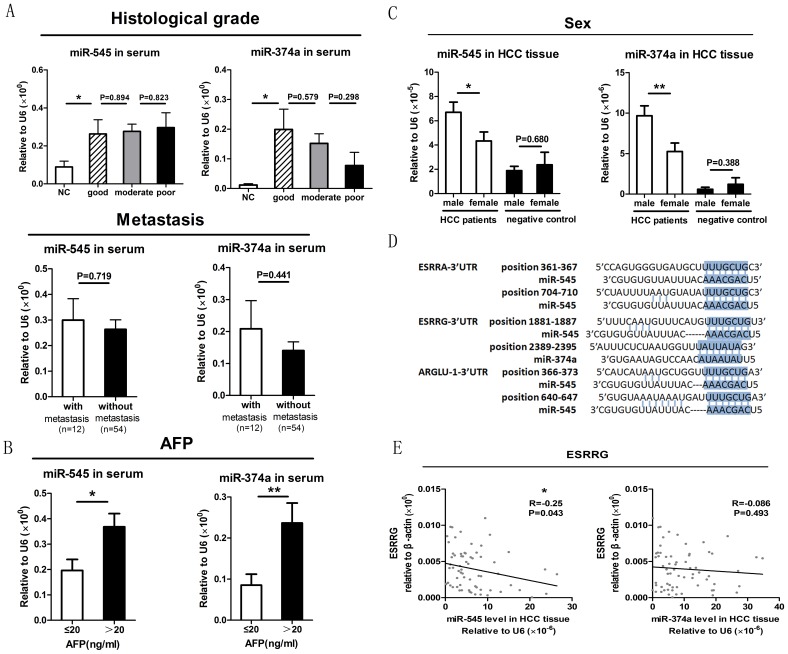
Correlation analysis of preoperative miR-545/374a serum expression with prognosis/diagnosis-related clinical features. (A) Correlation analysis of preoperative miR-545/374a serum levels with the prognosis-related clinical features of histological grade and metastasis. The histological grade was classified by pathological analysis as good, moderate or poor. Sera levels of miR-545/374a were analyzed by quantitative RT-PCR. The error bars in A represent mean ± Std. deviation. (B) Correlation analysis of preoperative miR-545/374a serum levels with the diagnosis-related clinical feature of AFP. The correlation analysis of CEA and γ-GT with the levels of miR-545/374a are shown in figure S1B. The error bars in B represent mean ± Std. deviation. (C) Gender-based investigation of Ftx microRNA cluster expression. HCC subjects and negative control subjects were grouped according to gender. MicroRNA 545 and miR-374a expression in HCC tissue was assayed by quantitative RT-PCR and compared between these groups. The error bars in C represent mean ± Std. deviation.*P<0.05, **P<0.01, t test. (D) Putative sex-related miR-545 and miR-374a target genes. The sequences comprising the miR-545 and miR-374a target sites are shown. (E) Correlation between HCC tissue miR-545/374a expression and ESRRG gene expression. The levels of miR-545/374a and ESRRG were monitored in 66 HCC tissue samples by quantitative RT-PCR. The correlation analysis of ESRRA and ARGLU-1 with miR-545/374a are shown in figure S1C. *P<0.05, Pearson's correlation test.

The serum levels of miR-545 and miR-374a were examined by quantitative RT-PCR of samples obtained from 66 HBV-HCC subjects and were correlated with prognosis/diagnosis related clinical features. Figure 5A shows that even though miR-545 and miR-374a in each histological grade show significant differences compared to the negative control, there were no statistically significant differences between any two histological grades. Figure 5A also shows that miR-545 and miR-374a showed no significant differences between patients with or without distal metastasis. Next, 3 previously established clinical features forecasting HCC recurrence (AFP, CEA, and γ-GT) were analyzed along with serum miR-545/374a expression (figure 5B, figure S1B). AFP positive patients (≥20 ng/ml) had a significantly higher miR-545/374a level than AFP negative patients (p = 0.012, p = 0.009, independent t test) (figure 5B). However, no significant differences in the levels of miR-545/374a were observed for patients expressing CEA or γ-GT (figure S1B).

### MicroRNA 545 and miR-374a are both higher in male HCC patients compared to female HCC patients, and ESRRG may be a target gene of miR-545

To investigate the potential role of miR-545/374a in HBV-HCC, the influence of gender was also evaluated (figure 5C). HCC subjects and negative control subjects were grouped according to gender. The levels of miR-545/374a were then monitored by quantitative RT-PCR of HCC tissue. The expression of both miR-545 and miR-374a were significantly higher in male vs. female HCC subjects (miR-545: male = 6.70, female = 4.34, p = 0.04; miR-374a: male = 9.68, female = 5.26, p = 0.008, independent t test). However, the distinction between male and female is not statistically significant in the negative control group (miR-545: male = 1.91, female = 2.19, p = 0.754; miR-374a: male = 0.37, female = 1.08, p = 0.273, independent t test).

The result is not surprising since the *Ftx* transcript is a positive regulator of *Xist*, the master regulator of the random X chromosome inactivation process [7]. Therefore the *Ftx* transcript may be responsible for a gender-based biological phenomenon. To explain the distinct expression between male and female HCC patients, we utilized in silico miRNA target prediction programs (TargetScan and miRanda) to analyze the potential target genes of miR-545 and miR-374a. The following 3 estrogen-related genes were found by this analysis: ESRRA, ESRRG and ARGLU-1. The sequence comprising the miRNA target sites for the 3 genes are shown in figure 5D. The levels of each gene were quantified in 66 HBV-HCC patients. Figure 5E shows the results obtained for ESRRG. Pearson's correlation showed that the ESRRG gene had a reverse correlation with the expression of miR-545 (r = −0.025, p = 0.043). No statistically significant correlations were observed between miR-545/374a and ESRRA or ARGLU-1 (figure S1C).

## Discussion

The *Ftx* gene gives rise to several RNA isoforms through a combination of alternative promoter usage, splicing and termination [7]. The miR-421/374b cluster and the miR-545/374a cluster are located in different introns and are controlled by distinct promoters. Since each microRNA cluster has distinct regulatory elements, it is possible that each cluster is differentially regulated during tumorigenesis. We found this to be the case in HBV-related HCC. Our findings indicated that the miR-545/374a cluster was significantly upregulated in HCC tissue, whereas the miR-421/374b cluster was not differentially regulated in HCC tissue. We then went on to further characterize the expression of the miR-545/374a cluster in HBV-related HCC.

**Figure 6:**
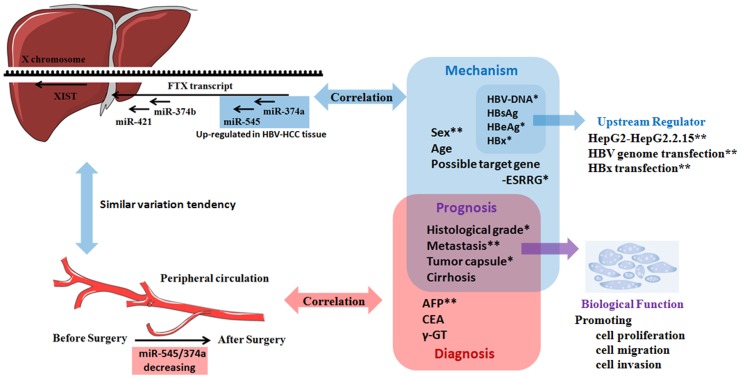
A consolidated model for the role of the miR-545/374a cluster in human HBV related hepatocellular carcinoma. Ftx gene encodes a lncRNA which harbors 2 cluster of microRNAs in its introns, the miR-374a/545 cluster and the miR-374b/421 cluster. In clinical specimens, the miR-545/374a cluster is upregulated in HCC tissue and is significantly correlated with prognosis-related clinical features. Serum miR-545/374a are tumor-derived, and their expression are significantly decreased after surgical excision of tumor tissue. MicroRNA 545 and miR-374a are both higher in male HCC patients compared to female HCC patients, and ESRRG may be a target gene of miR-545. In vitro studies, the miR-545/374a cluster promotes HCC cell proliferation, cell migration and invasion. Expression of miR-545/374a is positively regulated by HBV infection and may be induced by HBx expression.

We developed a model to describe the roles of the *Ftx* microRNA cluster in HBV-related HCC (figure 6) [13]. As the model shows, the expression of the miR-545/374a cluster is significantly upregulated in HCC tissue, and correlated with histological differentiation, incomplete tumor capsule formation and distal metastasis. *In vitro* cell transfection assays demonstrated that miR-545/374a may promote HCC cell proliferation, cell migration and invasion, thus implicating that overexpression of the miR-545/374a cluster may be responsible for tumor progression and poor prognosis of HCC patients. The correlation of miR-545/374a cluster expression with clinical HBV-related indices including HbeAg and HBV-DNA suggested that the miR-545/374a cluster is positively regulated by HBV infection. *In vitro* cell transfection studies confirmed these findings and showed that miR-545/374a expression may be induced by HBx expression. We also demonstrated that the levels of serum miR-545/374a are higher in HCC subjects compared to negative control patients. Furthermore, we found that serum miR-374a and miR-545 are tumor-derived, and showed a sharp drop in expression within 15 and 25 days after surgical tumor excision, respectively. We also determined that the expression of miR-545/374a correlated with AFP, a previously established clinical feature predicting HCC recurrence.

According to our studies, miR-545 and miR-374a have similar expression patterns and similar biological effects in HBV-related HCC. However, based on their sequence differences, the predicted target genes for each microRNA are different. A study in 2013 demonstrated that miR-374a regulates several target genes mediating the Wnt/β-catenin signaling pathway [8]. Our preliminary biological informative analysis showed that these genes may not be the most probable target genes of miR-545 (data not shown), further indicating that the target genes of miR-545 vs. miR-374a are different. Considering the possibility that miR-545 and miR-374a may have distinct mechanisms which promote tumor progression, we prefer to study them individually rather than studying the cluster as a whole.

From another perspective, now that miR-374a and miR-545 are transcribed off the same promoter, why their abundances are not always correlated (Figure 1C-F, Figure 5A)? It has been suggested that the expression of the intronic miRNAs may be coupled with that of the host mRNA, but differ from each other, especially in the pathological states. These observations point to diverse mechanisms and the existence of additional steps in the posttranscriptional processing of miRNA. Many of the RNA-binding proteins can bind to conserved loop sequences in the miRNA, and therefore, may form part of the gene regulatory network that influence the excision of miRNA in response to intra- or extra-cellular stimuli [14].

We first chose HCC tissue specimens to study the expression of miR-545/374a, and then developed a “from tissue to serum, from preoperative to postoperative” line of study to test the potential prognostic value of miR-545/374a using serum specimens. HCC is a serious condition associated with a high morbidity and mortality, and early detection is the key to effective surgical excision. Since the current biomarkers for HCC, including AFP, CEA and γ-GT, have limited sensitivity and specificity, new biomarkers are needed to improve the diagnosis and management of patients with liver pathologies. It is commonly believed that tumor-derived microRNAs can be released into the peripheral circulation and remain stable under harsh conditions due to protection from RNases [15]. Our results partially support that hypothesis. We found that serum miR-545/374a originated from tumor tissue, and the expression levels of miR-545/374a in HCC patients was higher than in negative control patients. Taken together these data support the hypothesis that serum miR-545/374a may be a potential diagnostic marker for HBV-related HCC. However, serum miR-545/374a may not be useful to predict the prognosis of HCC patients, because there was no significant correlation between serum miR-545/374a expression and a poor prognosis. In contrast, tissue miR-545/374a expression strongly correlated with a poor prognosis of HBV-related HCC patients. Therefore, miR-545/374a based prognosis-predicting may only be useful if tumor tissue is assayed.

We observed a gender-based difference in miR-545/374a expression. Specifically, male HCC patients had a significantly higher expression level of both microRNAs compared to female HCC patients. The distinction was not observed between normal male and female subjects. Based on these findings, we hypothesize that the comparatively small change in miR-545/374a expression in female HCC patients may serve as a protecting mechanism. Through biological analysis and correlation analysis, ESRRG was identified as a potential target gene of miR-545. The estrogen receptor-related receptor (ESRR) family are orphan nuclear receptors, which is closely related to the estrogen receptor family. They can bind to the estrogen response element. Previous researches have found ESRRA, a member of ESRR, can promote p53 gene expression and induce HCC progression in female patients [16], while whether ESRRG have a role in HCC is still unknown. Presently we are amplifying the sample size, especially the female HCC patients. Respective study in female and male patient may better indicate the potential role of ESRRG. Elucidation of the mechanism of miR-545 mediated regulation of ESRRG expression requires further investigation.

## Conclusions

In summary, the expression of the miR-545/374a cluster located in the lncRNA *Ftx* is significantly upregulated in HBV-related HCC tissue, and correlated with a poor prognosis of HCC patients. Serum miR-545/374a originated from tumor tissue, and potentially may be utilized as a novel screening and diagnostic marker for HCC occurrence in routine clinical practice. ESRRG is a potential target gene of miR-545 and we hypothesize that this mechanism may be related to the low incidence of HBV-HCC in females, which requires further investigation.

## Supporting Information

Figure S1 **MicroRNA expression data and different clinical features.** (A) No significant correlation is showed concerning patients' age (years, Mean±Std.deviation, 52.73±9.712; Median, 53). Pearson's correlation. (B) HBV-HCC patients are divided into positive and negative groups based on their serum CEA and γ-GT level, however no statistical significance is showed. Independent t test.
(TIF)

Figure S2 **Wound healing assay: miR-374a/545 may increase the migration of HCC in vitro.** The cells in six-well plate were scratched with pipette tip, incubated at 37°C, 5% CO_2_ for 24 hours after being transfected by miroRNA mimics or inhibitor, then monitored by photographing for wounding. Result showed that, cell transfected with miR-374a or 545 mimics exhibit higher migration ability, while inhibition of miR-374a or miR-545 notably delayed healing of wound and inhibited cell migration on the surface of the tissue culture plate.
(TIF)

Table S1 **List of the oligonucleotides used in this study.**
(DOCX)

Table S2 **A. The expression of microRNAs in** ***Ftx*** **transcript in human tissues. B. The expression of miR-374a/545 in human serum.**
(DOCX)
