# Supplementary figures and images for: The miR-545/374a Cluster Encoded in the Ftx lncRNA is Overexpressed in HBV-Related Hepatocellular Carcinoma and Promotes Tumorigenesis and Tumor Progression
Source: PLoS One. 2014 Oct 9;9(10):e109782. doi: 10.1371/journal.pone.0109782 (PMC4192320; doi:10.1371/journal.pone.0109782)

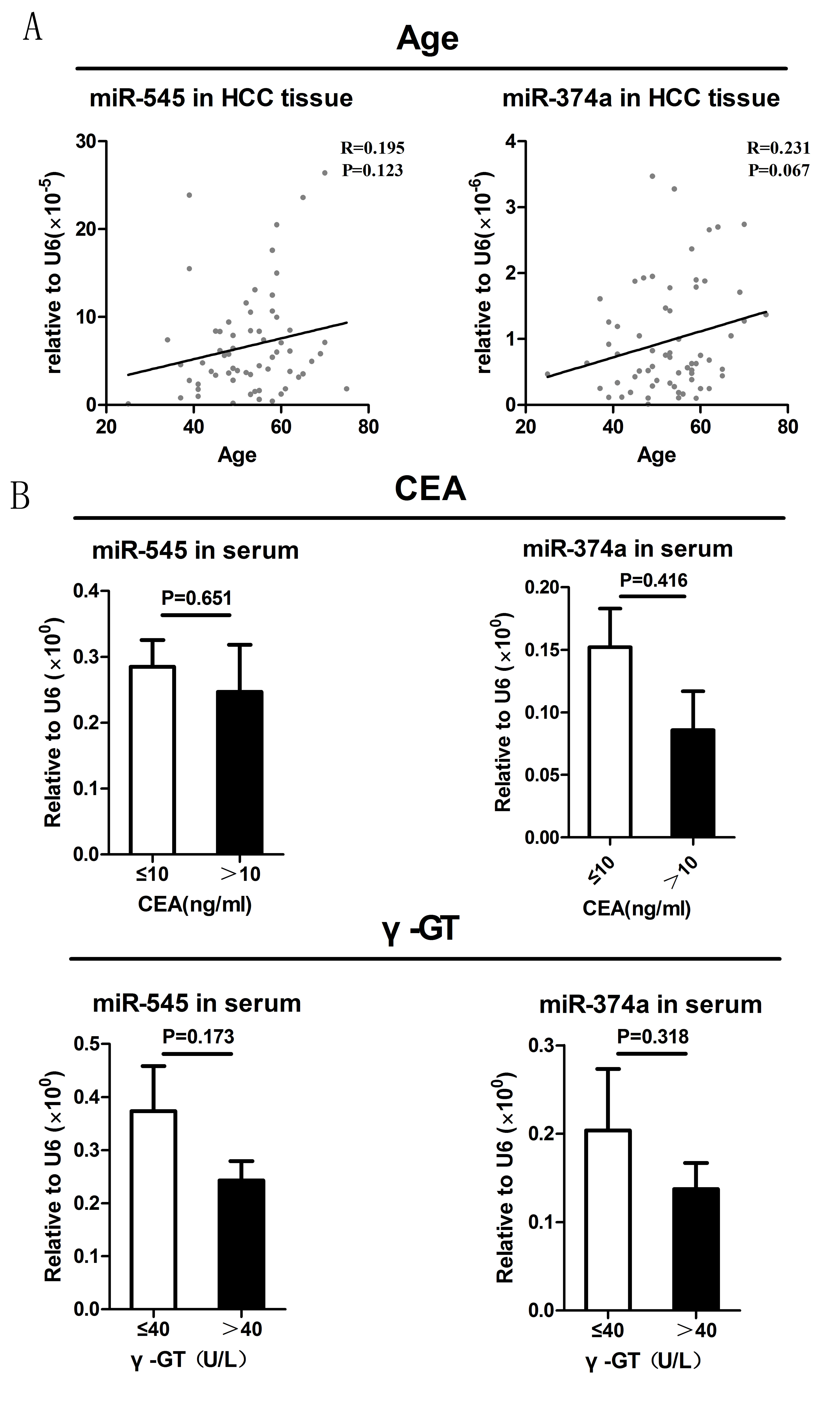

Supplement: Figure S1 — MicroRNA expression data and different clinical features. (A) No significant correlation is showed concerning patients' age (years, Mean±Std.deviation, 52.73±9.712; Median, 53). Pearson's correlation. (B) HBV-HCC patients are divided into positive and negative groups based on their serum CEA and γ-GT level, however no statistical significance is showed. Independent t test. (TIF) [file pone.0109782.s001.tif]

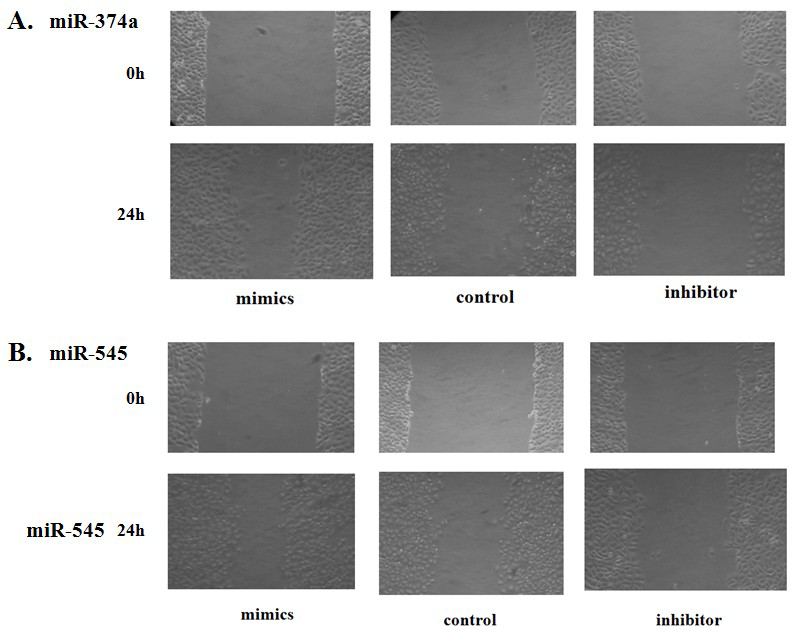

Supplement: Figure S2 — Wound healing assay: miR-374a/545 may increase the migration of HCC in vitro. The cells in six-well plate were scratched with pipette tip, incubated at 37°C, 5% CO2 for 24 hours after being transfected by miroRNA mimics or inhibitor, then monitored by photographing for wounding. Result showed that, cell transfected with miR-374a or 545 mimics exhibit higher migration ability, while inhibition of miR-374a or miR-545 notably delayed healing of wound and inhibited cell migration on the surface of the tissue culture plate. (TIF) [file pone.0109782.s002.tif]
