# Supplementary material for: The miR-545/374a Cluster Encoded in the Ftx lncRNA is Overexpressed in HBV-Related Hepatocellular Carcinoma and Promotes Tumorigenesis and Tumor Progression
Source: PLoS One. 2014 Oct 9;9(10):e109782. doi: 10.1371/journal.pone.0109782 (PMC4192320; doi:10.1371/journal.pone.0109782)
Supplement: Table S1 — List of the oligonucleotides used in this study. (DOCX) [file pone.0109782.s003.docx]

**Table S1.** List of the oligonucleotides used in this study.

Name^a^  Sequence( 5’ 3’)

**microRNA and corresponding mimic, inhibitor**

miR-374a UUAUAAUACAACCUGAUAAGUG

miR-374a mimic (sense) UUAUAAUACAACCUGAUAAGUG

miR-374a inhibitor CACUUAUCAGGUUGUAUUAUAA

miR-545 UCAGCAAACAUUUAUUGUGUGC

miR-545 mimic (sense) UCAGCAAACAUUUAUUGUGUGC

miR-545 inhibitor GCACACAAUAAAUGUUUGCUGA

miR-374b AUAUAAUACAACCUGCUAAGUG

miR-421 AUCAACAGACAUUAAUUGGGCGC

**Primers for mRNA RT-PCR**

HBx F CAGGCAACTATTGTGGTTTCACAT

R ATAAGATAGGGGCATTTGGTGGT

Wnt5a F CATCCTCATGAACCTGCACAA

R AGCCAGCATGTCTTCAGGCTAC

ESRRA F CTACCACTATGGTGTGGCATCCTG

R TCCGCTTGGTGATCTCACACTC

ARGLU-1 F GAAGCCAAACGCATCATGGA

R TTTGGCTTGTGCTTCTGCAAT

Wnt5b F TTCTGACAGACGCCAACTCCT

R GCCATGTGCTCCTGGTACAAT

ESRRG F  TGATGAAACATCAACCCAGTGCTA

R GGGATGTGCCAATTTCTGAACTC

β-actin F GGGAAATCGTGCGTGACTTAAG

R TGTGTTGGCGTACAGGTCTTTG

^a^ F, forward primer; R, reverse primer.
