# Supplementary material for: The miR-545/374a Cluster Encoded in the Ftx lncRNA is Overexpressed in HBV-Related Hepatocellular Carcinoma and Promotes Tumorigenesis and Tumor Progression
Source: PLoS One. 2014 Oct 9;9(10):e109782. doi: 10.1371/journal.pone.0109782 (PMC4192320; doi:10.1371/journal.pone.0109782)
Supplement: Table S2 — A. The expression of microRNAs in Ftx transcript in human tissues. B. The expression of miR-374a/545 in human serum. (DOCX) [file pone.0109782.s004.docx]

**Supplement Table 2A.** The expression of microRNAs in *Ftx* transcript in human tissues.

|  | | | | tissue | Mean | Std. error | Median | P |
| --- | --- | --- | --- | --- | --- | --- | --- | --- |
| miR-374a | | HCC | | | 9.73*10^-6 | 1.016*10^-6 | 7.035*10^-6 |  |
|  | NT | | | | 1.05*10^-6 | 0.21*10^-6 | 0.3415*10^-6 | 0.000** |
|  | NC | | | | 0.73*10^-6 | 0.34*10^-6 | 0.3477*10^-6 | ＞0.05 |
| miR-545 | HCC | | | | 6.60*10^-5 | 0.722*10^-5 | 4.874*10^-5 |  |
|  | NT | | | | 3.27*10^-5 | 0.367*10^-5 | 2.500*10^-5 | 0.003** |
|  | NC | | | | 2.06*10^-5 | 0.414*10^-5 | 1.920*10^-5 | 0.034* |
| miR-374b | | | HCC | | 0.444 | 0.208 | 0.00522 |  |
|  | NT | | | | 0.206 | 0.166 | 0.000221 | ＞0.05 |
|  | NC | | | | 0.724 | 0.314 | 0.026 | ＞0.05 |
| miR-421 | HCC | | | | 0.2454 | 0.08158 | 0.01446 |  |
|  | NT | | | | 0.2761 | 0.08301 | 0.009175 | ＞0.05 |
|  | NC | | | | 0.1371 | 0.04515 | 0.06715 | ＞0.05 |

HCC，hepatocellular carcinoma; NT, nontumor tissue; NC, negative control.

**Supplement Table 2B.** The expression of miR-374a/545 in human serum.

|  | serum | Mean | Std. error | Median | P |
| --- | --- | --- | --- | --- | --- |
| miR-374a | NC | 0.01198 | 0.00389 | 0.00279 |  |
|  | HCC-before surgery | 0.1528 | 0.02688 | 0.072 | ＜0.01** |
|  | HCC-after surgery | 0.08265 | 0.01867 | 0.025 | 0.042* |
| miR-545 | NC | 0.08864 | 0.0295 | 0.01704 |  |
|  | HCC-before surgery | 0.2561 | 0.03136 | 0.1265 | ＜0.05* |
|  | HCC-after surgery | 0.135 | 0.01856 | 0.0635 | 0.001** |

HCC, hepatocellular carcinoma; NC, negative control.
